# *PLOS Neglected Tropical Diseases* 2018 Reviewer and Editorial Board Thank You

**DOI:** 10.1371/journal.pntd.0007252

**Published:** 2019-02-27

**Fig 1:**
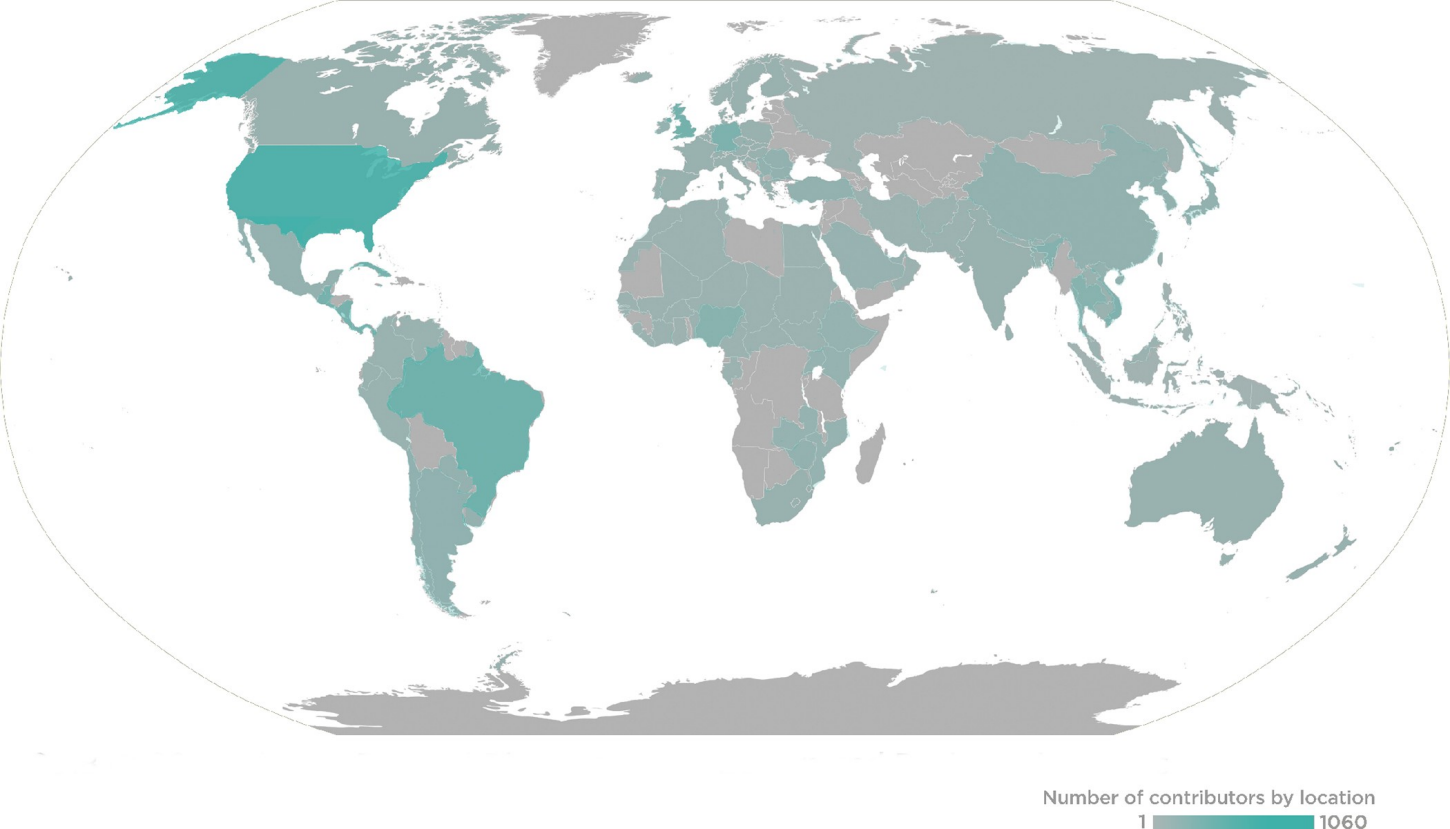
2018 *PLOS Neglected Tropical Diseases* Global Editor and Reviewer Locations.

PLOS and the *PLOS Neglected Tropical Diseases* team want to sincerely thank all of our Editorial Board Members, Guest Editors, and Reviewers for the journal in 2018. Your contributions of time and expertise support your research community, advance scientific progress, and continue to make *PLOS Neglected Tropical Diseases* a leader in its field. This past year, *PLOS Neglected Tropical Diseases* received the assistance of 260 Editorial Board members, 204 Guest Editors, and 2,473 Reviewers, who handled 1,749 manuscripts that resulted in 811 publications (Fig 1).

We’re deeply grateful to all of our volunteers whose dedicated efforts support *PLOS Neglected Tropical Diseases* and Open Science. Thank you all for your work!
